# Multifaceted Aspects of Metabolic Plasticity in Human Cholangiocarcinoma: An Overview of Current Perspectives

**DOI:** 10.3390/cells9030596

**Published:** 2020-03-03

**Authors:** Mirella Pastore, Giulia Lori, Alessandra Gentilini, Maria Letizia Taddei, Giovanni Di Maira, Claudia Campani, Stefania Recalcati, Pietro Invernizzi, Fabio Marra, Chiara Raggi

**Affiliations:** 1 Department of Experimental and Clinical Medicine, University of Florence, 50134 Florence, Italy; mirella.pastore@unifi.it (M.P.); giulia.lori@unifi.it (G.L.); alessandra.gentilini@unifi.it (A.G.); marialetizia.taddei@unifi.it (M.L.T.); giovanni.dimaira@unifi.it (G.D.M.); claudiacampani.cc@gmail.com (C.C.); fabio.marra@unifi.it (F.M.); 2 Department of Biomedical Sciences for Health, University of Milan, 20133 Milan, Italy; stefania.recalcati@unimi.it; 3 Division of Gastroenterology and Center for Autoimmune Liver Diseases, San Gerardo Hospital, Department of Medicine and Surgery, University of Milan-Bicocca, 20900 Monza, Italy; pietro.invernizzi@unimib.it

**Keywords:** cholangiocarcinoma, mitochondria, OXPHOS, PGC-1α, tricarboxylic acid cycle, fatty acids, fatty acid synthase

## Abstract

Cholangiocarcinoma (CCA) is a deadly tumor without an effective therapy. Unique metabolic and bioenergetics features are important hallmarks of tumor cells. Metabolic plasticity allows cancer cells to survive in poor nutrient environments and maximize cell growth by sustaining survival, proliferation, and metastasis. In recent years, an increasing number of studies have shown that specific signaling networks contribute to malignant tumor onset by reprogramming metabolic traits. Several evidences demonstrate that numerous metabolic mediators represent key-players of CCA progression by regulating many signaling pathways. Besides the well-known Warburg effect, several other different pathways involving carbohydrates, proteins, lipids, and nucleic acids metabolism are altered in CCA. The goal of this review is to highlight the main metabolic processes involved in the cholangio-carcinogeneis that might be considered as potential novel druggable candidates for this disease.

## 1. Introduction 

Cholangiocarcinoma (CCA), first identified by Steiner and Higginson [1] belongs to a heterogeneous group of malignancies that occur at any point of the biliary tree [2]. It can generate from epithelial cells in the biliary tracts (ie cholangiocytes) and peribiliary glands [3] and, possibly, from progenitor cells or even hepatocytes [4]. Based on the anatomical site, CCAs is classified in two major groups: intrahepatic (iCCA) and extrahepatic (eCCA) CCA [5]. The iCCA is located in the intrahepatic biliary tree [5,6] whereas eCCA may rise in the extrahepatic bile ducts [7,8]. CCA is characterized by very poor outcome, showing an increase in incidence and mortality rate worldwide [9,10,11]. Potentially curative surgical treatment is restricted to the small subset of patients with early stage disease (approximately 35%). At present, the available medical therapeutic treatments for advanced or metastatic CCA have limited beneficial efficacy [12]. 

Several risk factors, related to chronic biliary inflammation and/or cholestasis, such as primary sclerosing cholangitis, have been associated with higher risk of CCA development [13]. Chronic hepatitis B and C, cirrhosis, alcohol excess, smoke, obesity and diabetes are mainly associated with the development of CCA [9,14]. Several epidemiological reports have shown an increase in global occurrence (up to 10 folds) and mortality for iCCA, whereas the incidence for eCCA remained either unchanged or slightly decreased [15,16,17,18,19]. CCA is mainly diagnosed in an advanced clinical stage of the disease, when curative treatment is generally unsuccessful [20]. This is in part due to the lack of clinical manifestation of the tumor in the early stage, especially in case of iCCA, and in part to the absence of specific and sensitive biomarkers. To reduce global mortality from cholangiocarcinoma, efforts must be multifaceted and focus on prevention, early identification of high-risk individuals and prompt diagnosis as well as molecular based targeted therapies for established disease [14].

In this scenario, the concept of tumor metabolism is worth particular interest for several reasons: (i) metabolic modification is a well-known hallmark of cancer, (ii) oncogenes drive alterations in cancer metabolism, (iii) metabolites can control gene and protein expressions, and (iv) metabolic proteins and/or metabolites represent diagnostic and prognostic biomarkers [21,22,23,24,25]. Indeed tumour cells have unique metabolic and bioenergetic features, allowing them to survive in poor nutrient environments and maximize cell growth [22]. Besides glucose metabolism, many other different metabolic pathways that involve carbohydrates, proteins, lipids and nucleic acids are deregulated in cancer cells. Furthermore, tumour cells are able to shift between different metabolic pathways depending on their energetic needs, environmental stress and other factors. Due to the high heterogeneity in the metabolic pathways that occur in different tumors, and even inside the same tumour [26], this review will highlight the main metabolic processes that occur in CCA progression.

## 2. Glucose Metabolism and CCA

The first reported example of a reprogrammed metabolic pathway in cancer is the Warburg effect or aerobic glycolysis [27,28] by which tumour cells mainly convert pyruvate to lactate through glycolysis even when oxygen is abundant [26]. The increase in glycolytic flux allows glycolytic intermediates to supply secondary pathways in order to satisfy the metabolic demands of proliferating cells [27]. Warburg effect is in large part induced by the transcription factors hypoxia inducible factor 1 alpha (HIF-1α) and c-Myc but also, in lesser extent, by other signaling pathways, such as the phosphatidylinositol 3-kinase (PI3K)-Akt mammalian target of rapamycin (mTOR) signaling, and the activation of oncogenes and inactivation of tumor suppressors [26].

Recently, it has been recognized that Warburg effect in cancer cells is not a general phenomenon. Indeed, it has been described also a “reverse” Warburg effect, by which tumour cells induce oxidative stress in surrounding stromal fibroblasts, that activates aerobic glycolysis with production of high levels of lactate, which is exported out and recaptured by the tumor. On turn, cancer cells convert the lactate back into pyruvate, which can be used in the tricarboxylic acid (TCA) cycle to fuel oxidative phosphorylation. This theory of “lactate shuttle” between cancer and stromal cells proposes the involvement of the stroma in influencing tumour metabolism [29,30,31,32]. 

A strict dependence of CCA cells on glucose is proved by several papers reporting an increase in the expression levels of the glucose transporter GLUT-1 associated to this disease. Recently Kubo Y. et al., described that the survival of patients with GLUT-1–positive tumors were significantly poorer than those of patients with GLUT-1–negative ones. Moreover, GLUT-1 silencing was able to decrease migration and invasion of CCA cells [33]. Coherently, also Ikeno Y et al., observed a significantly poorer survival of patients with high GLUT-1 expressing CCA with respect to patients with low GLUT-1 expression [34]. Other authors suggest that CCA cells are heavily dependent on glucose consumption. Suzuki H. et al., showed that GLUT-1 expression correlates with 18F-2-fluoro-2-deoxy-d-glucose uptake in the primary iCCA. Furthermore, high numbers of GLUT-1-positive cells in the tumor samples were found in patients with poorly differentiated carcinoma, confirming a correlation between GLUT-1 expression and histological differentiation [35] (Table 1).

Along these lines, Saengboonmee C. et al., [66] clearly reported a direct link between high glucose levels in culture media and CCA cell aggressiveness, in term of increased rates of cell proliferation, adhesion, migration and invasion with respect to CCA cells cultured in normal glucose. Indeed, high glucose levels stimulated both the phosphorylation and the nuclear translocation of the signal transducer and activator of transcription 3 (STAT3). In turn, STAT3 up-regulated the transcription of downstream target genes associated with an aggressive phenotype, including cyclin D1, vimentin and matrix metalloproteinase-2. Moreover, tumor tissues from CCA patients with diabetes mellitus showed increased levels of phospho-STAT3, confirming the link between high glucose patient blood and STAT3 activation. In agreement, glucose reduction or STAT3 inhibition reduced the proliferative effect on CCA cells [66]. More recently, the same authors reported the effects of high glucose concentrations on O-GlcNAcylation protein expression and metastatic potentials of CCA cells. Indeed, Phoomak C. et al., [67] described a marked increase of migration and invasion of highly metastatic CCA cell lines when cultured in high glucose medium. In this condition, high glucose uptake may support glucose flux through the hexosamine biosynthesis pathway (HBP) to produce uridine diphospho-N-acetylglucosamine, a substrate for glycosylation, such as O-GlcNAcylation. The flux of the HBP can be regulated by the expression of the rate-limiting enzyme, glucosamine-fructose-6-phosphate amidotransfrase (GFAT), whose levels are upregulated in CCA cell lines maintained in high glucose condition and in tissue samples from patients correlated with high levels of O-GlcNAcylated proteins. Interestingly, both O-GlcNAcylation of vimentin and its stability were increased in CCA cells cultured in high glucose and correlated with improved migratory abilities. Conversely, administration of a GFAT inhibitor suppressed glucose-induced O-GlcNAcylation, vimentin expression and reversed tumor cell invasion [67]. 

In keeping with an amplified flux of glycolysis, several evidences suggest an increase of lactate levels, the final product of the Warburg metabolism, in CCA. Indeed, increased expression of LDH-A, the nicotinamide adenine dinucleotide (NADH)-dependent enzyme that catalyses the conversion of pyruvate to lactate, was associated with shorter survival of CCA patients [68]. In line, a study conducted by Yu Y et al., showed that lactate dehydrogenase A (LDH-A) was overexpressed in 52 of 54 (96%) paraffin-embedded cancer tissues from iCCA patients. Moreover, LDH-A silencing inhibited cell growth and promoted HUCCT1 cell death [69].

The pentose phosphate pathway (PPP), that directs glucose flux to produce nicotinamide adenine dinucleotide phosphate (NADPH) and ribose-5-phosphate, is crucial in CCA metabolism. Qu X et al., [36] demonstrated that, in human CCA QBC939 cells, cisplatin resistance was linked to a high ability to uptake glucose, to generate lactate and to increase the activity of PPP. Indeed, PPP stimulation can be related to an improved flow of autophagy and to an increase in the antioxidant capacity of QBC939 cells, sustaining drug resistance. In line, autophagy inhibition, strongly decreased glucose-6 phosphate dehydrogenase activity, the first enzyme of PPP, and reduced both the NADPH/NADP and reduced glutathione/oxidized glutathione ratio in QBC939 cells. These events caused a drop in the antioxidant capacity of QBC939 cells and a consequent burst of intracellular reactive oxygen species (ROS), leading to an increased sensitivity to cisplatin treatment [36] (Table 1).

Other studies support the up-regulation of Warburg metabolism in CCA: pyruvate dehydrogenase kinases (PDKs) are Ser/Thr kinases that phosphorylate/inactivate mitochondrial pyruvate dehydrogenase, thus decreasing the oxidation of pyruvate in the tricarboxylic acid (TCA) cycle and increasing the Warburg effect [70]. Sanmai S. et al., [37] demonstrated that PDK1, PDK2 and PDK3, were significantly overexpressed in 15 CCA tissues compare to normal ones. Moreover, PDK3 expression levels were considerably higher in CCA sera compared with the benign biliary diseases and healthy groups. In agreement, PDK3 levels correlated with a short survival time in CCA [37]. Overall, these data suggest that inhibition of mitochondrial metabolism and hence the sustenance of the glycolytic flux can be fundamental in CCA growth.

Also Xu L., et al., pointed out a central role of PDK regulation in CCA: in this manuscript the authors demonstrated that in CCA the decrease of SIRT3 expression induced the glycolytic flux through the hypoxia inducible factor α (HIF1α)/PDK1/pyruvate dehydrogenase (PDHA1) axis, promoting tumor progression. In particular, SIRT3, a NAD+ dependent deacetylase, inhibited the Warburg effect through HIF1α deacetylation/destabilization. Indeed, it is known that lysine acetylation is able to regulate the stability of several proteins, including HIF1α. Reduced HIF1α stability inhibits glycolysis and down-regulates PDK1. The authors showed that in CCA cells, as well as in both tissue samples and in a xenograft model, SIRT3 was downregulated and the glycolysis enhanced. Conversely, the induction of SIRT3 was able to efficiently reduce tumor proliferation via an anti-Warburg effect mediated by the HIF1α/PDK1/PDHA1 pathway [38] (Table 1). 

Overall these data highlight a marked increase in the glycolytyic flux and in lactate production of CCA cells with respect to benign ones. The enhancement of these metabolic pathways may be instrumental for cancer cells to acquire epithelial to mesenchymal transition (EMT) markers for tumor progression as well as to sustain divergent pathways, such as PPP, in order to guarantee both biosynthetic intermediates to mantain cell proliferation and the antioxidant capacity to counteract oxidative stress [36,66,67]. 

Despite these data showing a marked dependence of on Warburg metabolism, Dan Li et al., demonstrated that PGC1α overexpression reversed the effect and increased pyruvate flux into the mitochondria by up-regulating both pyruvate dehydrogenase E1 alpha 1 Subunit and mitochondrial pyruvate carrier 1 expression. These events induced mitochondrial biogenesis and the metabolic switch to oxidative phosphorylation (OXPHOS), finally facilitating CCA cells migration and invasion [49]. Collectively all these data suggest a great ability of CCA cells to shift between different metabolic pathways in order to adapt to environmental changes and to survive. Probably the achievement of an OXPHOS metabolism is mandatory to acquire a more aggressive phenotype of CCA cells, facilitating the maintenance of stemness features and the improvement of the metastatic abilities. Thus, we may suppose that Warburg metabolism and OXPHOS contribute at different times or on distinct subpopulation during CCA progression. This evidence could also explain the conflicting data concerning the use of Metformin, an inhibitor of the mitochondrial respiratory chain (complex I) and hence of OXPHOS, with the onset/development of CCA.

In particular, it has been observed that Metformin significantly reduced the risk of iCCA in diabetic patients by 60% [71,72]. In keeping, also Jiang X. et al., proved that in a xenograft tumor model, Metformin significantly inhibited cell growth in vivo, resulting in decreased tumor volume and weight [73]. Moreover, CCA patients treated with Metformin showed reduced tumor size and improved postoperative survival [73]. On the contrary, Yang Z. et al., [74] have shown that Metformin, even if it is able to reduce cancer cell proliferation and to induce cell cycle arrest, did not improve the survival of CCA patients with diabetes [75].

Thus, further studies are still required to clarify whether Metformin as well as other metabolic drugs could be successfully used in clinic for CCA treatment. 

## 3. Lipid Metabolism in CCA

The largest part of normal tissues obtains lipids through the uptake of free fatty acids (FFAs) and lipoproteins from the bloodstream. Nevertheless, it has been described that many cancers showed a reactivation of *de novo* lipid synthesis, together with an increased expression of several key enzymes [76,77,78,79,80] similarly to embryonic tissue.

However, the role of fatty acid synthase (FASN) in the liver is different in HCC and CCA development. Li et al., [81] have demonstrated that in hepatocellular cell lines, FASN silencing strongly affected proliferation rate, together with apoptosis increase [81]. Moreover, in a hydrodynamic injection mouse model FASN downregulation totally abrogated AKT-dependent hepatocarcinogenesis [81], thus FASN plays a key role in HCC development. Nevertheless, in human and mouse iCCA tissues FASN expression was down- regulated respect to non-tumor adjacent tissues. Indeed, it has been observed that in AKT/Ras mice, which develop both HCC and iCCA, FASN knocking-down prevented only HCC onset [51,82] (Table 1). 

Although lipid metabolism is pivotal for tumor development, CCA seems to be totally independent on *de novo* fatty acids (Fas) synthesis [81]. Indeed Li et al., [81] have further analyzed the involvement of exogenous FAs uptake. They demonstrated how the deprivation of lipoprotein in culture media highly inhibited CCA cells growth [81]. In addition, the expression levels of FAs transporters such as cluster of differentiation 36 (CD36) and solute carrier family 27 member 1 (SLC27A1) were upregulated in AKT/notch-intracellular domain liver tumor tissues. These results were confirmed in human iCCA samples, where SLC27A1 was overexpressed respect to normal tissues. Moreover, SCL27A1 silencing in both HUCCT1 and HuH28 cell lines led to a decrease of cells growth [52]. This reduction of CCA cells proliferation also synergized with FASN knocking down [81] (Table 1). 

Many evidences have demonstrated that FAs are actively transported through cell membrane by specific proteins, the fatty acid transport proteins (FATPs). In the liver, the main proteins involved in this transport are FATP2, FATP1 and FATP5, the fatty acid binding proteins (FABP1, FABP4, and FABP5), and the translocase CD36. In particular, FABP5 seems to play distinct role in iCCA and eCCA. A recent research suggests that FABP5 upregulation characterizes eCCA, reflecting its worse prognosis respect to iCCA. This difference may be due to distinct embryological tissues origin and histological location during carcinogenesis [53] (Table 1). 

Recently, it has been demonstrated that adipocytes contribute to EMT, invasion, proliferation and progression, in several cancer types [83,84,85,86,87]. Nie et al., [88] found that co-culture with adipocytes led CCA cells to express mesenchymal biomarkers overexpression and cell-to-cell adhesion alteration. The acquisition of these mesenchymal markers in CCA cells could be due to adypocite-derived FAs. In fact, they showed that the adipocyte-derived FABP4 mediated migration, invasion and lipid accumulation in CCA, by shifting FAs between adipocytes and cancer cells [88]. 

Even sphingolipids, phospoinositides and eicosanoids derive from FAs. Eicosanoids are generated from arachidonic acid that is converted into prostaglandin H_2_ by cyclooxygenases (COX1 and COX2) [89]. Recent evidences have demonstrated that prostaglandins (PG) play a key role in CCA onset. In fact, in CCA cells and pre-cancerous bile duct lesions has been observed an up-regulation of COX-2 levels respect to normal bile duct cells [54,55,56,57,58]. In addition, in CCA cells COX-2 overexpression increases PGE_2_ production, promoting tumor growth [90,91], whereas COX-2 down-regulation with molecular or pharmacological techniques lowers PGE2 release and prevents cancer development and invasion, both in vitro and in vivo tests [55,58,90,91,92,93,94]. Most of the cellular functions depend on lipids availability, thus lipid biosynthesis is strictly regulated to avoid lipid toxicity and membrane dysfunction [95,96].

Importanly, sphingosine-1-phosphate is a pivotal regulator of cell proliferation and survival. The enzyme sphingosine kinase (SPHK) converts the sphingolipid sphingosine to S1P, regulating cell fate. It has been demonstrated that the isoform 1 of SPHK is involved in tumor proliferation, angiogenesis and transformation [97,98]. Chen et al. [99] identified SPHK1 overexpression as a marker of poor prognosis for iCCA. They demonstrated that the inhibition of SPHK1 with SK1-I induces apoptosis in CCA cell lines, together with growth arrest. Moreover SK1-I intraperitoneal injection in CCA xenograft mouse model, leads to a significant suppression of tumor growth [99]. This preclinical study has provided a rationale for clinical trials in CCA patients. ABC294640 is an inhibitor of SPHK2 (Ki = 9 µM, 3.4 µg/mL) that depletes S1P promoting autophagy and/or apoptosis in tumor cells [100,101,102]. Based on its strong preclinical profile, a first-in-human phase I trial was undertaken to analyze the drug’s safety, and to identify the maximum tolerated dose. This study was conducted in 22 patients with solid tumors. Interestingly, the best outcome was a partial response in CCA patients [103] (Table 2).

In addition, statins are a class of drugs that inhibiting HMG-CoA reductase, lead to a reduction of serum cholesterol. Several studies have demonstrated that statins are also able to inhibit growth and induce apoptosis in several cancer cells. Buranrat et al., [119] showed that Simvastatin and Atorvastatin exert an antiproliferative action by inhibiting HMG-CoA reductase and p21 activation. Moreover, these statins induced CCA cell death acting through caspase 3 and cytochrome c, inhibited cell migration and reduced colony formation ability of CCA cell lines [119] (Table 2). All these data suggest that statins could be also used for cancer chemoprevention or chemotherapy [8].

## 4. Protein Metabolism and CCA

Usually cancer cells metabolize glucose to produce ATP through aerobic glycolysis but they have to use other energy sources such as glutamine to satisfy fast proliferation [122].

Glutamine is a non-essential ammino-acid, which plays a critical role in cell growth and proliferation acting in the synthesis of other non-essential amino acids, in the modification of chromatin, in the anti-oxidative defense, in the synthesis of nucleotides as nitrogen donor and in the refueling of the TCA cycle intermediate (anaplerosis) [59]. While the role of anaplerosis has been demonstrated in HCC its role in CCA is unknown [60] (Table 1).

Due to continuous loss, replenishment of TCA intermediates is necessary in cancer cells, and this fact causes increased glutamine consumption. A reduction in extracellular glutamine concentration has been demonstrated in most malignancies and it seems to have a role on the cell susceptibility to different apoptosis triggers. Indeed cells starving glutamine are more sensitive to Fas ligand, tumor necrosis factor-α (TNF-α) and heat shock-mediated apoptosis [122].

The role of glutamine has been studied also in CCA. A paper evaluating glutamine level in CCA and the effects of its deprivation on therapeutic response has been recently published [59]. The study demonstrated a strong depletion of glutamine in the tumor core region of the majority of the samples and this reduction was found to induce a cessation of proliferation or cell death (in vitro addiction to glutamine). In a later part of the study a gradual reduction of external glutamine concentrations has been evaluated. This part of the experiment suggested that eCCA cells (EGI-1 and TFK-1) could proliferate under long-term glutamine withdrawal overcoming their addiction to glutamine. Moreover, this study demonstrated that double-deprivation stress through cyclic hypoxia and nutrient starvation eliminates hypoxia-induced chemoresistance to cisplatin thanks to a reduced c-Myc expression [59].

Several transporters mediate the passage of glutamine across the plasma membrane and mitochondrial inner membrane. L-type amino acid transporter 1 (LAT1), a Na^+^-independent neutral amino acid transporter, is overexpressed and plays a critical role in various human cancers, including CCA [61] (Table 1). For this reason, LAT1 has been evaluated as a target of CCA treatment.

Janpipatkul et al., demonstrated that KKU-M213 cells (CCA cells derived from Thai patients with iCCA) treated with 2-aminobicyclo-(2,2,1)-heptane-2-carboxylic acid had a reduction in L-leucine uptake that consequently inhibits mTOR pathway activity and therefore reduces cell proliferation and viability. Similar results were obtained with LAT1 knockdown KKU-M213 cells [62] (Table 2).

Yothaisong et al., in another work demonstrated the role of JPH203 in inhibiting LAT1 transport activity. This compound suppressed amino acid uptake and CCA cell growth through altering the cell cycle distribution patterns and inducing apoptosis [63] (Table 1 and Table 2).

Arginine is a semiessential amino acid synthesized from citrulline in two steps: argininosuccinate synthetase (ASS) converts citrulline and aspartate in argininosuccinate that is later converted to arginine by argininosuccinate lyase. 

Argininosuccinate synthetase deficiency has been demonstrated in various malignancies such as melanoma, prostate cancer, pancreatic cancer, renal cell carcinoma, HCC and CCA [120]. 

Tumor cells that have low ASS expression are unable to synthetise arginine and must depend on extracellular arginine. On the basis of this knowledge, it has been hypothesized that a reduction of arginine in the surrounding tumor cells could lead to a reduction in cell proliferation. ADI-PEG 20 is a cloned arginine-degrading enzyme (arginine deaminase) conjugated with polyethylene glycol that degrades arginine to citrulline. The role of ADI-PEG 20 has been evaluated in patients with advanced HCC with good results in a phase II trial, whereas the phase III trial did not met its primary endpoint [121] (Table 2). ADI-PEG20 has been studied also in patients with CCA where the treatment significantly inhibited growth of RmCCA-1 and HuCCA cells by decreasing both the percentage of viable cells and the proliferative activity in a dose-dependent manner [120]. 

Arginine is also a substrate of the urea cycle, a metabolic process that occurs exclusively in the liver. The urea cycle transforms ammonia in urea that is less toxic for the organism. Various studies have shown that the urea cycle is altered both in HCC and CCA. A suppression of members of the urea cycle, probably due to epigenetic alterations, has been demonstrated in HCC [123].

Changes in the expression and metabolism of other amino acids compared to healthy patients or patients with other malignancies have also been reported in CCA.

Murakami et al., demonstrated that a multi-omics approach, integrating metabolomic and transcriptomic datasets, was able to distinguish between iCCA and HCC tumours. Regarding amino acids metabolism four amino acids (Lys, Pro, Leu and IIe) were more diversely expressed in iCCA/iCCA-non tumor than in HCC and HCC-non tumor [124].

## 5. CCA and Iron 

Iron is necessary for cellular replication and growth, so it is an essential element for life. However, excess iron can facilitate the formation of the most reactive and toxic forms of oxidants through the Fenton reaction [125]. The regulation of cellular iron homeostasis is due to different proteins: the transferrin receptor (TfR1), responsible for transferrin-bound iron up-take, major players in ferroportin (Fpn), the only cellular iron exporter, and ferritin, the iron store protein [126]. An association between high body iron content and cancer in the general population has been demonstrated [127], as well as positive relation between increased body iron stores and the risk of liver cancer [128] (Table 1). 

It has been shown also a relationship between liver iron and CCA. In particular, it was demonstrated that high expression of TfR1, with consequent iron uptake, contributes to CCA progression and poorer clinical outcomes [64] (Table 1). Accordingly, it has been showed that high ferritin expression in epithelial cells from CCA patients is a negative prognostic index (6) (Table 1). Moreover, in line with these findings, it has been found significantly reduced Fpn mRNA levels, that means reduced iron release, in tumor cells of CCA patients sample compared to matched surrounding liver, suggesting that elevated iron content is a negative prognostic factor [65] (Table 1). 

In general, the alterations of iron trafficking in cancer cells lead to iron acquisition or decrease iron release (the high iron needs of tumor cells to sustain cell proliferation) and it has been shown that these alterations of cellular iron metabolism are dependent on the direct action of tumor suppressors and oncogenes [129].

Recently, it has also been shown that increased iron retention in CCA-cancer stem cells (CSCs) could be a novel metabolic factor involved in CCA growth [65]. In particular, it has been shown that CSCs have a phenotype symptomatic of elevated cellular iron content and oxidative stress; increased iron levels are accompanied by important changes in the expression of stemness markers, such as EMT markers, in these cells that can be reversed by iron removal. This finding was mirrored by data showing a trend toward shorter survival in CCA patients with iron levels.

Finally, it has to be noted that CCA-CSCs, despite higher levels of iron and ROS, seem to be less susceptible to the ferroptosis, a recently characterized mechanism which causes cell death through iron-dependent ROS production and lipid peroxidation [130], than CCA cells. This finding could be perhaps due to the well-known CSC resistance to various types of chemoterapeutic agents. 

All the observations reported here regarding CCA and iron seem to suggest a hopefully translation into an effective adjunct therapeutic approach based on iron deprivation that should be provided.

## 6. Molecular Aspects Underlying CCA Metabolism

**Figure 1:**
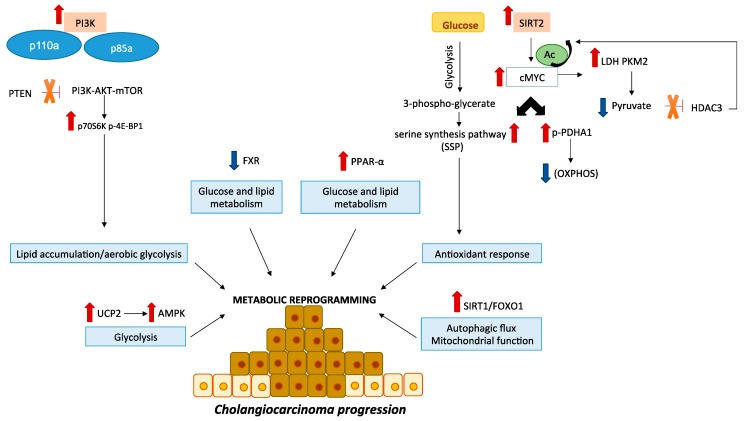
Crucial signaling pathways in CCA metabolism. The PI3K/AKT/mTOR pathway is an essential modulator of cell metabolism, growth and survival in CCA. Activating mutations of gene coding for the p110a and p85a subunits of PI3K and loss of the phosphatase and tensin homolog (PTEN) have been reported in CCA. Moreover, it has been shown the overexpression of downstream mTOR effectors, such as p70S6K and p-4E-BP1. SIRT2/cMYC pathway is overexpressed in CCA cell lines and mediates the activation of the PDHA1 by inhibition the OXPHOS as well as the activation of SSP that acts against ROS production. During CCA progression increase lactate levels while pyruvate levels decrease. Moreover, cMyc induces an increase of LDH and PKM2 expression, which afterwards reduces pyruvate levels. Being the pyruvate an HDAC3 inhibitor, a reduction of its levels removes HDAC3 inhibition and promotes cMyc overexpression. Sirt1/FOXO1 pathway is involved in autophagy and mitochondrial functions. UCP2 promotes CCA development through AMPK pathway. FXR expression is downregulated in human tissues and CCA cell lines inducing tumor progression by modulating cellular metabolism. PPAR-α is overexpressed in CCA patients and modulates several genes involved in glucose and lipid metabolism as well as immune responses. All these alterations induce the activation of pathways that promote CCA progression through a reprogramming of cellular metabolism.

In recent years, an increasing number of studies have elucidated how metabolism is altered in cancer cells, based on observations that components of signal transduction pathways frequently regulate cell metabolism (Figure 1). Tumor cells select mutations that enhance signal transduction through pathways that converge upon a set of metabolic processes that contribute to tumorigenic process. The rewiring of cancer cells metabolism includes abnormalities in glucose homeostasis and other nutrients such as aminoacid and lipid and impairment of mitochondrial function [22]. 

### 6.1. PI3K-AKT-mTOR Signaling Pathway 

The signaling pathway PI3K-AKT-mTOR is one of the most frequently altered pathways in human cancer [131] and plays a critical role in tumorigenesis through the modulation of cellular metabolism, including glucose homeostasis, nutrients uptake and utilization. Its deregulated activation supports enhanced cancer cell growth and proliferation and drives tumor initiation and progression. The role of PI3K/AKT/mTOR pathway in human CCA has been assessed both in vitro and in vivo. The most common alterations include activating mutations and overexpression of the PI3K genes (*PIK3CA* and *PIK3R1)* that encode respectively for the p110a and p85a subunits [132]. PI3K is a lipid kinase that phosphorylates the 3′-hydroxyl group of the inositol ring of phosphatidylinositol to generate 3′-phosphoinositides [39]. Uncontrolled activation of this kinase causes accumulation of transit signals responsible for pathway triggering. Protein overexpression and functional activation of PI3K have been associated with tumor progression, differentiation, nodal involvement and reduced overall survival (OS) [40]. Positive immunostaining for unphosphorilated and phosphorylated form of AKT have been reported in human CCA specimens, with higher rates in neoplastic cells compared to the surrounding normal tissue [40]. Increased *mTOR* gene copy number and elevated phospho-mTOR levels have been described in biliary cancer specimens in comparison to the adjacent normal or dysplastic epithelium [41]. Moreover, several studies reported overexpression of downstream mTOR effectors in CCA. For example, high p70S6K expression has been associated with poor tumor differentiation [41] and high p-4E-BP1 level has been correlated with poor prognosis in a CCA patient’s cohort [41]. The lipid phosphatase and tensin homologous (PTEN) is responsible for negative regulation of the pathway and inactivating mutations or deletion of PTEN lead to uncontrolled activation of PI3K/AKT/mTOR signaling. In fact, PTEN loss has been related to poor tumor differentiation, nodal involvement and shorter survival in CCA [42] (Table 1). Finally, several growth factor receptors have been involved in cholangiocarcinogenesis via PI3K/AKT/mTOR pathway. For example, EGFR/HER2-dependent PI3K/AKT phosphorylation has been demonstrated in CCA cell lines and human CCA tissue samples [133,134]. The PI3K/AKT/mTOR pathway acts downstream of insulin growth factor receptor 1 (IGFR1) in CCA tissues. Indeed IGFR1 inhibitors NVP-AEW541 [135] and BMS-536924 prevented AKT activation and exerted antiproliferative effects in CCA cells and in a xenograft CCA mouse model [136]. 

The studies described in this paragraph showed mutations, gene copy number alterations and aberrant protein phosphorylation of the key players of this pathway in CCA. These alterations lead to PI3K/AKT/mTOR pathway activation, which in turn triggers anabolic processes such as mRNA translation, protein synthesis and post-translational modulation of metabolic enzymes and switch to aerobic glycolysis, supporting enhanced growth, proliferation, chemoresistance.

Pre-clinical studies demonstrated that the following PI3K inhibitors, LY294002, Buparlisib (BKM120) and PI-103 have significant in vitro activity against CCA cells. 

LY294002 inhibited cell proliferation, invasiveness and EMT [111] by decreasing p-AKT and p70S6K levels. 

Buparlisib demonstrated important anti-proliferative activity in either mutant or wild-type KRAS CCA cells [112], while the inhibitor PI-103 affect cell proliferation in a xenograft CCA mouse model [113]. Clinical reports on the activity of PI3K inhibitors in CCA are limited. A Phase I trial evaluated the activity of mFOLFOX6 (modified Folinic acid/5-Fluorouracil/Oxaliplatin) combined with Buparlisib in 17 patients with advanced refractory gastrointestinal tumors (4 of whom were CCAs). The maximum tolerated dose (MTD) (primary endpoint) of Buparlisib was established at 40 mg. Only 8 patients out of 17 were treated for at least two cycles (8 weeks) and were evaluable for response. At the end of this study the authors concluded that the combination of mFOLFOX6 with Buparlisib resulted in increased toxicity compared to either treatment alone and did not prove efficacious in treatment of advanced refractory biliary tract malignancies ([114], Table 2).

In addition, Copanlisib is a pan-class I inhibitor of PI3K with predominant PI3K-α/δ activity with clinical activity and manageable safety when administered as monotherapy in a phase II study. Kim and colleagues showed the results of a phase I study to determine the safety, tolerability and recommended phase II dose of Copanlisib in combination with Gemcitabine or with Cisplatin plus Gemcitabine (CisGem) in patients with advanced solid malignancies, including CCA [115].

Concerning AKT inhibitors the allosteric inhibitors (MK2206) or adenosine triphosphate (ATP)-competitive molecules proved to be effective to impair cell proliferation, survival and in different CCA cell lines [137,138].

MK2206 as an orally administered single agent was evaluated at the weekly dose of 200 mg in a Phase II study enrolling 8 patients affected by advanced, pretreated CCAs. None of the 8 patients had an objective response and only two patients achieved stable disease (25%) at 12weeks ([116], Table 2). 

The pathway of mTOR was assessed in vitro using an mTOR inhibitor, Everolimus, showing dose-dependent decrease of cell proliferation by this molecule [139]. A phase I study reported that Everolimus achieved 50% disease-control-rate in a subgroup of 22 advanced CCA patients ([104], Table 2). Subsequently a phase II study, in which patients with advanced CCA were enrolled, used Everolimus as a treatment option reporting an OS of 9.5 months [105]. At the same time in a phase II Italian study with Everolimus 39 patients with advanced and pre-treated CCA had progression-free survival (PFS) of 3.2 months, and OS of 7.7 months [106] while an Australian phase II study with Everolimus, conducted on patients with advanced CCA, showed objective response rate of 12 % and PFS of 6.0 months. Recently in 27 unselected patients, Everolimus displayed clinical activity as first-line monotherapy in advanced CCA with PFS of 5.5 months and OS of 9.5 months [105]. In another phase II study that aimed to evaluate the activity of Everolimus in 10 patients with *PIK3CA* amplification/mutation or *PTEN* loss refractory solid cancer, only one patient with CCA with *PTEN* loss experienced disease control ([107], Table 2). Concerning other mTOR inhibitors, Rizell reported a cohort of Sirolimus used in patients with HCC (*n* = 21) and iCCA (*n* = 9). Three (33%) of nine patients with iCCA achieved stable disease after Sirolimus treatment [108]. In a clinical study enrolling patients with PIK3CA mutant/amplified refractory solid cancer, Sirolimus failed to demonstrate the clinical benefit in a patient with hilar CCA (PIK3CA E545K mutation) who experienced disease progression following the second cycle of Sirolimus with PFS of 0.9 months ([109], Table 2). Regarding clinical trials using mTOR inhibitors and standard treatment, the only published study was performed to determine the MTD of different combinations of Everolimus and chemotherapeutic agents ([110], Table 2). 

Despite several studies showed encouraging results more extensive investigations are necessary to elucidate the efficacy of mTOR inhibitors in the treatment of CCA.

### 6.2. SIRT2/cMYC Signaling Pathway

Recently, another pathway, sustained by a divergence of the glycolytic flux, has been described to have a crucial role in promoting CCA proliferation. It is known that the glycolytic intermediate 3-phospho-glycerate can be shunted to the serine synthesis pathway (SSP) to sustain an antioxidant response. Indeed, a metabolic reprogramming of CCA cells towards the production of anti-oxidant molecules may support cancer cell growth through the reduction of tumor oxidative stress [43]. Xu L. et al., [43] observed that the histone deacetylase sirtuin 2 (SIRT2) and its downstream target cMYC, were overexpressed both in two human CCA cell lines (HUCCT1 and RBE) with respect to normal bile duct epithelial cells and in 48 CCA samples compared to adjacent tissues. The SIRT2/cMYC pathway is able to reprogram CCA metabolism through: i) phosphorylation of the PDHA1 and hence inhibition of OXPHOS; ii) activation of SSP to counteract ROS production by increasing antioxidant defenses, thus protecting CCA cells from oxidative stress-induced apoptosis. Indeed, CCA patients with elevated expression levels of SIRT2, cMYC, and p-PDHA1 were associated with lower survival rates [43]. The same authors showed that CCA cells stabilized a positive feedback loop sustaining Warburg metabolism [43]. At this regard, it has been demonstrated that CCA cells, through an avid consumption of glucose, decreased the level of pyruvate in favor of increased levels of lactate. Actually, tumour cells induce a MYC-mediated increase of LDH and Pyruvate kinase isoenzyme M2 expression levels causing a decrease in pyruvate levels. Pyruvate is an inhibitor of the histone deacetylase HDAC3, which deacetylases cMYC at the K323 site, promoting its stabilization. Thus cMYC, by decreasing pyruvate levels, removes HDAC3 inhibition and sustains a positive feedback loop. This circuitry protects cancer cells from apoptosis [140]. Moreover, the high levels of carbon flux supply the increased production of metabolic intermediates, giving advantages in a challenging tumor microenvironment [141,142,143]. 

### 6.3. Uncoupling Protein 2 (UCP2)

Yu J et al. [44] have produced another recent paper supporting the association of CCA with glycolysis. They showed that the uncoupling protein UCP2, promoting the proton leak to decrease the electrochemical potential and hence ATP synthesis, was up-regulated in human CCA and this event was associated with a worse prognosis. Up-regulation of UCP2 sustains the EMT and cell invasion. Indeed, in in vitro experiments they proved that inhibition of UCP2 suppressed cell proliferation and migration, reversed EMT, and reduced drug resistance as well as spheroid formation. Interestingly, all these events correlated with reduced glycolytic flux, reinforcing the idea that UCP2 contributes to the progression of CCA through a glycolysis-mediated mechanism [44] (Table 1).

### 6.4. Transcription Factor FOXO1

Autophagy plays a key role in the maintenance of cellular homeostasis. In healthy cells its homeostatic activity represents a robust barrier against malignant transformation and control of autophagy progression in cancer cells is an effective strategy to halt tumor growth [144]. Impaired autophagy could interfere with key cellular processes including energy metabolism control and mitochondrial dysfunction [145]. In CCA cell line QBC939, the authors demonstrated that the transcription factor FOXO1 regulated both basal and serum starvation-induced autophagy via the interaction between Acetylated-FOXO1 and Atg7. FOXO1 silencing or Sirt1 inhibition, affected autophagic flux, impaired mitochondrial function and induced apoptosis in a cholangiocarcinoma cell line, suggesting a role of Sirt1/FOXO1 pathway in the interplay between autophagy and mitochondrial dysfunction in CCA [50] (Table 1).

### 6.5. Nuclear Receptors: Farnesoid X Receptor (FXR) and Peroxisome Proliferator Activated Receptor-α (PPAR-α)

The nuclear receptors have bi-functional activities, are capable of binding hormone as well as directly activating gene transcription. Nuclear receptors and their coregulators exhibit critical functions in the regulation of metabolic processes and they are central regulator of feeding and fasting state [146]. 

Among nuclear receptors, farnesoid X receptor (FXR) regulates bile acid homeostasis and it is activated in the fed state to exert a direct effect on metabolic pathways, including suppression of both gluconeogenesis and lipogenesis [147,148].

Rather, peroxisome proliferator-activated receptor-α (PPAR-α) is a well-known inducer of hepatic fatty acid oxidation in the fasted state and it promotes gluconeogenesis [149].

FXR and PPAR-α have opposite functions in metabolic mechanisms, that determine opposite effects on gluconeogenesis, indicating extensive functional interactions and conflicting metabolic effects.

The high expression of FXR was observed in tissues exposed to high bile acid concentration, including cholangiocytes, and this type of nuclear receptor is also involved in several processes such as lipid and glucose metabolism, fibrosis, and regeneration [150,151,152,153].

Moreover, Erice et al., [45] have recently shown a downregulation of FXR expression in both human tissues and CCA cell lines. They have found that the FXR agonist, obeticholic acid (OCA), inhibited the orthotopic CCA tumor growth in immunodeficient mice and this was associated with decreased expression of proliferation markers such as Ki67 and PCNA. Moreover, in vitro it has been demonstrated that OCA inhibited CCA cell proliferation and migration and these inhibitory effects of OCA on CCA cells were associated with decreased mitochondrial energy metabolism [45] (Table 1).

Loss of FXR activity could be an important molecular event in the initiation and progression of CCA, and the downregulation of FXR expression could promote cancer development, modulating the energy metabolism of CCA cells. 

A further fundamental factor in CCA progression is PPAR-α. In a recent study, there have been identified endogenous RNAs which modulated the expression of target genes, which could have a role in tumor occurrence and progression in patients with CCA. The analysis performed has revealed that the genes identified were strongly linked to metabolism. Pathway analysis has found that carbon metabolism, bile secretion, fat digestion, and PPAR signaling pathway were processes significantly enriched and played a pivotal role in CCA patients [46] (Table 1).

Recently, through the meta-analysis of CCA that represents a new systematical multivariate analysis tool, there have been identified 25 genes and five pathways associated with type 2 diabetes, obesity, and dyslipidemia. All five pathways were associated with the metabolism of lipids. Among these pathways, there was PPAR-α signaling which represents a significant modulator of numerous genes involved in glucose and lipid metabolism as well as inflammatory processes. PPAR-α in human liver determines also the down-regulation of several genes involved in various immunity-related pathways [47] (Table 1). 

### 6.6. Isocitrate Dehidrogenase (IDH) 

Isocitrate dehidrogenase enzymes play an important role in the TCA cycle, catalysing the oxidative decarboxylation of isocitrate to α-ketoglutarate, producing carbon dioxide and NADPH [26]. Frequent mutations in the IDH1 and IDH2 genes have been shown in different types of cancer, including CCA [154,155]. IDH mutations (mIDH) are known as neomorphic mutations, since they give a new function to the product of the altered gene. Indeed, they establish a pathway for the NAPDH-dependent conversion of the wild-type IDH product, α-ketoglutarate into 2-hydroxyglutarate (2-HG) [156] with a significant decrease in NADPH production [157] (Table 1). 2-HG is normally present as two enantiomers, dextrogyre (*D*) and laevogyre (*L*). Interestingly mIDH preferably provokes an increase in the *D*-enantiomer of 2-HG rather than the L-enantiomer and these forms can be measured in the serum by gas chromatography coupled to mass spectrometry [158] or liquid chromatography combined with mass spectrometry [159,160].

In the most mIDH, the wild-type IDH function is lost due to mutation of critical amino acid residues in the catalytic domain, IDH1 R132 and IDH2 R172, which normally binds the b-carboxyl group of isocitrate to start catalysis [156,161]. 

In CCA, the most common mutations of IDH1 are R132C (more frequent), R132G and R132L, which are distinct from the R132H mutation found in gliomas [155,162,163]; nevertheless, these activating alterations have been shown to increase the serum level of 2-HG in cholangiocarcinoma [160]. The prevalence of IDH2 mutations in CCA is instead R172K, R172M, and R172G [162].

Accumulating evidence identifies 2-HG as an oncometabolite, since it interferes with many regulatory pathways involving α-ketoglutarate-dependent dioxygenases, including those implicated in epigenetic remodeling and DNA repair [164,165,166]. Indeed 2-HG hinders the function of enzymes that use α-ketoglutarate as a co-enzyme, including histone and DNA demethylases, by competitively occupying the same pockets as α-ketoglutarate [167,168]. 

Genomic studies reported that mutations in IDH1/2 were found in approximately 10% (range, 5-36%) of iCCA, whatever the geographical area of the world [154,169,170,171] while mutations in IDH1 were linked to iCCA with a prevalence of about 20% in North America [162]. 

The histological characteristics of CCA mIDH1 tumors are not well defined: they seem to vary from small duct types and unique geographic type fibrosis [172], to a large spectrum of histopathology features [155]; the discordancy could be due to the relatively small number of analyzed patients for each single study. Intriguingly, mIDH CCA tumors present definite molecular characteristics, including low levels of chromatin modifiers, upregulation of mitochondrial genes, as components of the TCA cycle and electron transport chain, and increased copy numbers of mitochondrial DNA [155]. Of note, mIDH hypermethylates ARID1A promoter can lower the chromatin remodeler expression [155]. A recent study on a large cohort of of iCCA patients (n=496) based on occurrence of mutations in IDH, KRAS and TP53 genes, revealed unique mutational, structural and epigenetic features, which could facilitate prediction of therapeutic sensitivity for each iCCA patient, by evaluating individual genotyping [173]. It has been reported that mIDH occured mostly in woman with a percentage of about 60-70%, a higher incidence than in the whole CCA population [174], and that three genes resulted often co-mutated with mIDH: ARID1A (22%), BAP1 (15,5%) and PBRM1 (13,3%) [174]. 

Unfortunately none of the studies on mIDH have showed significant association between the presence of these alterations and clinopathological features (OS, PFS or time of progression) [174]. 

The high incidence of IDH mutations in numerous malignant tumors has promoted the development of specific inhibitor compounds resulting in several clinical trials. 

AG-120 (Ivosidenib), an oral selective inhibitor of mIDH1 characterized by good tolerance, has displayed therapeutic activity with reduction of 2-HG levels in various solid tumors, including CCA [117]. In CCA patients treated with Ivosidenib, 6-month PFS was 38% and 12-month PFS was 20%; 56% of patients achieved stable disease and 5% achieved a partial response. 

A follow-up trial in mutated IDH1 CCA patients that are not eligible for curative resection, transplantation, or ablative therapies prior to enrollment, is underway (NCT02989857) with completion date on 11/01/2019. Patients with IDH2 mutated solid tumors have been enrolled in a phase I-II clinical trial evaluating the orally bioavailable compound AG-221, an inhibitor of mutant IDH2 (NCT02273739) [118] (Table 2).

Very recently, it has been reported that IDH1 mutation in CCA enhanced the formation of intrahepatic biliary organoids (IBOs) and sped up the glucose uptake and glucose metabolism as well as upregulation of some metabolytes in TCA cycle (citrate, fumarate and malate). Interestingly platelet isoform of phosphofructokinse-1 (PFKP), a rate-limiting glycolytic enzyme, was also upregulated and knockdown of its gene reduced IBOs formation, indicating that the induction of glycolysis in mutant IBOs could occur by enhancing the expression of this enzyme. A direct link between the IDH 1 mutation and the expression of PFKP was demonstrated by the decrease of the protein level in mutant IBOs treated with AGI-5198 (a mIDH1 inhibitor) and the increase of the protein level in mutant IBOs treated with 2-HG (Table 2). In addition, mutant IBOs could sustain survival in ATP depletion conditions by obtaining ATP through the activation of AMPK. These results suggest that IDH mutation drives two-way metabolic rewiring status [48]. (Table 1).

### 6.7. Tumor Suppressor p53

In addition to oncogenes, tumor suppressors, like the p53 transcription factor, can regulate cancer metabolism [175]. The p53 canonical activity is related to cell cycle arrest, senescence, apoptosis and DNA repair, but recently it has been demonstrated that p53 results also involved in regulating cancer metabolism and oxidative stress [176,177]. Indeed loss of this protein causes an increased glycolytic flux to induce anabolism and redox balance that ultimately leads to tumorigenesis [175].

The p53 protein-encoding gene *TP53* is mutated or deleted in 50% of all human cancers [22]. Particularly in iCCA, while commonly harbors mutations affect *IDH1/2, BAP1, KRAS, TP53, SMAD4, and ARID1A* [178,179]. The p53 inactivation is the most common dysfunction (observed in 37% of iCCAs) [180,181], recently revealed as predictor of a poor prognosis [182]. The p53 is also involved in eCCA development and serum antibody against this protein was reported to be useful for the early detenction of this form of cancer [183,184]. Of note *TP53* loss enhanced reprogramming of hepatocytes to biliary cells promoting CCA, pointing at p53 as a critical regulator of this process [185]. 

### 6.8. Hif-1α Signaling Pathway

Nowadays, hypoxia is widely recognized as a critical tumor-promoting player. Hypoxia exerts a selective pressure on cancer cells, promoting the clonal expansion of the most aggressive tumor phenotypes. Cellular responses to hypoxia are primarily mediated by the activation of HIFs, i.e., helix-loop-helix transcription factors that bind to specific DNA sequences known as hypoxia-response elements. HIFs consist of two subunits: α (HIF-1α, HIF-2α, or HIF-3α) and β (HIF-1β). Among HIF-α isoforms, HIF-1α is the best characterized, due to its ubiquitous expression. HIF-1α is a master regulator of transcriptional response to low oxygen and it is associated with resistance to radiotherapies and chemotherapies and poor clinical outcome [186]. HIF-1α promotes the transcription of several target genes involved in key cellular processes including glucose metabolism [187].

In CCA specimens, HIF-1α is typically overexpressed compared to bile ducts of peritumoral areas [188], and its high expression was reported as an independent prognostic factor for overall and disease-free survival [189] (Table 1). Consistently, in vitro studies clearly demonstrated that hypoxia-induced signaling led CCA cells to gain a more malignant phenotype. For instance, low-oxygen culture conditions, as well as treatment with cobalt chloride (i.e., a chemical inducer of HIF-1α), promoted a substantial increase in CCA cell motility and invasiveness [190,191]. In a recent study the authors found that the hepatocyte growth factor (HGF) antagonist NK4 is effective on inhibiting CCA cell hypoxic growth and invasion both in vitro and in vivo. In particular, NK4 inhibited HIF-1α expression and HIF-1-induced CCA cells invasion in vitro and tumor angiogenesis in vivo, suggesting an involvement of HGF/MET signaling on the CCA malignant phenotype promoted by HIF-1α [192]. Moreover HIF-1α regulated the expression of Rab1a via miR-212-3p. Rab1a is a member of RAS oncogene family and its overexpression activates mTORC1 signaling that plays a critical role in tumor metabolic reprogramming [193]. It has been demonstrated that Rab1a was overexpressed in iCCA tissues and associated with poor prognosis of iCCA patients and knockdown expression of HIF-1α under hypoxia condition decreased the expression of Rab1a expression while miR-212-3p was increased. Suppression of Rab1a led to lower proliferation rate and migration ability both in vitro and in vivo by inhibiting cell cycle and EMT. In conclusion this study demonstrates that HIF-1α/miR-212-3p/Rab1a axis, a well-known mTORC1 associated signalling, enhances proliferation and migration of CCA cells [194].

It has been described that HIF-1 is able to stimulate lipid accumulation through HIF-2 protein induction, a factor implicated in neutral lipids deposition into lipid droplets [195]. In addition, in the liver HIF2 is involved in changing of lipid metabolism in response of VHL lack. The specific liver-deletion of VHL in mice lead to steatosis, together with increased lipid droplets formation and βoxidation down-regulation [196]. The exact role of these lipid droplets in tumor development and survival is not fully understood, but it has been proposed that the storage of triacylglycerides could be useful in intermittent hypoxia conditions, since they can be utilized as a quickly available energy source after reoxygenation [96]. Although FAs utilization is crucial for cancer cells survival in hypoxic condition, indeed it seems that tumor cells preserve a certain grade of ß-oxidation, as shown by CPT1C requirement [197]. In addition, it has been demonstrated that hypoxia also stimulated FASN expression, through Akt and SREBP1. To regulate FA biosynthesis, hypoxia is able to alter lipid composition of tumor cells, by impairing lipid synthesis and modification pathways on one hand and modifying exogenous lipids uptake on the other. In fact, a recent study showed how hypoxic tumor cells undertake lysophospholipids from the environment to meet the requirement of unsaturated FAs [198].

## 7. Future Directions

Several studies have shown that impaired metabolism in cancer cells may result in the generation of an unbalanced metabolically microenvironment [199]. In particular, the metabolic effects on immune cell phenotype have recently become of major interest [200,201,202].

Rising evidence have shown that crucial feature of immune cells such as activation, differentiation, as well as regulation of immune responses and immunological memory are strictly associated to their metabolic state. For instance, metabolism modulates macrophage polarization and consequently their functions. Conventionally, M1 macrophages mediate an inflammatory and antitumor response through glycolysis and reduced mitochondrial activity. Conversely, anti-inflammatory M2 macrophages show enhanced fatty acid oxidation and high OXPHOS as well as more mitochondria [203,204]. In M1 macrophages, glycolysis favours the introduction of the carbon flux into the oxidative PPP pathway, which produces NADPH for the generation of ROS via NADPH oxidases [205]. 

Further, M2 macrophages express specific changes in some metabolic pathways such as arginine metabolism that is moved toward the production of ornithine and polyamine by arginase I and II instead of citrulline and NO by inducible nitric oxide synthase (iNOS) [205,206].

Moreover, fatty acid oxidation supports the pro-tumor potential of M2 macrophages and provides a crucial energy source for M2 macrophage polarization. [202]. Additionally, it has been observed that the inhibition of fatty acid oxidation in tumor-associated macrophages promotes antitumorigenic differentiation and inhibits tumor growth [207].

Furthermore several findings indicate a key role of glycolysis in Treg proliferation in the tumor setting. Intra-tumoral Tregs show a high glucose uptake [208] and they have high levels of GLUT-1 on the cell surface, express high levels of glycolysis-related genes, and engage a higher glycolytic flux as measured in terms of extracellular acidification rate directly compared to effector T cells [209]. These evidences suggest that glycolysis may be involved in the maintenance and proliferation of tumor- infiltrating Tregs. 

CCA growth is deeply affected by a multitude of immune cells. However, the consequences of the metabolic programming of immune cells on CCA progression are largely indeterminate and should be further studied in order to develop more effective combinatorial therapies.

Owing to the increasing focus of research on immune-modulating therapy options, knowledge of the molecular mechanisms underlying immune infiltration of CCA is of growing interest. The development of innovative approaches based on generating T cell products for immunotherapy requires the acquiring of new knowledge on metabolic aspects that favors anti-tumor function of T cells. 

Overall, the emerging interfaces between metabolism and immune response: “immunometabolism” may allow identifying metabolic pathways that may modulate the immune response during CCA initiation and progression.

## Figures and Tables

**Table 1:** Major altered metabolism pathways in cholangiocarcinoma.

Metabolic Pathways	Metabolic Target	Effects on Cholangiocarcinoma (CCA)	Reference
Glucose metabolism	GLUT-1 upregulation	Correlation with in vitro CCA cell invasion. Correlation with larger tumor size, poor differentiation, metastasis and poor prognosis of CCA.	[33,34,35]
	PPP upstimulation	Sustainment of both the antioxidant capacity of CCA cells and cisplatin resistance.	[36]
	PDK overexpression	High serum PDK3 levels correlate with short survival of patients with CCA. PDK1 expression promotes glycolysis and CCA cell proliferation.	[37]
	SIRT3 effects mediated by HIF1α/ PDK1/PDHA1 pathway	Decrease of SIRT3 expression induced the glycolytic flux through the hypoxia inducible factor α (HIF1α)/PDK1/ PDHA1 axis, promoting CCA progression.	[38]
	Deregulation of PI3K-AKT-mTOR signaling	Protein overexpression and activation of PI3K have been associated with tumor progression, differentiation, nodal involvement and reduced OS. Upregulation of activated form of AKT have been reported in neoplastic cells compared to the surrounding normal tissue; Increased *mTOR* gene copy number and elevated phospho-mTOR levels have been described in biliary cancer specimens in comparison to the adjacent normal or dysplastic epithelium. PTEN loss has been related to poor tumor differentiation, nodal involvement and shorter survival in CCA.	[39,40,41,42]
	SIRT2 overexpression	SIRT2 and its downstream target cMYC, were overexpressed both in human CCA cell lines and in 48 CCA samples compared to adjacent tissues, The SIRT2/cMYC pathway is able to reprogram CCA metabolism through inhibition of OXPHOS and activation of SSP to counteract ROS production, thus protecting CCA cells from oxidative stress-induced apoptosis.	[43]
	UCP2 overexpression	Up-regulation of UCP2 sustains the EMT and cell invasion of CCA cells.	[44]
	FXR downregulation	Initiation and progression of CCA, and the downregulation of FXR expression could promote cancer development, modulating the energy metabolism of CCA cells.	[45]
	PPAR-α upregulation	Tumor occurrence and progression in CCA patients with.	[46,47]
	IDH1 and IDH2 mutations	Increase of glucose uptake and glucose metabolism as well as upregulation of some metabolites in TCA cycle. Upregulation of PFKP.	[48]
Mitochondrial metabolism	PGC1α upregulation	Promotion of CCA metastasis both in vitro and in vivo.	[49]
	Sirt1/FOXO1 stimulation	Involvement in autophagy and mitochondrial dysfunction in CCA cells.	[50]
Lipid metabolism	FASN down-regulation	In human and mouse iCCA tissues FASN expression was down- regulated respect to non-tumor adjacent tissues.	[51]
	FA transporter (SLC27A1) overexpression	SCL27A1 silencing in CCA cell lines led to a decrease of cells growth.	[52]
	FA transporter (FABP5) over expression	Correlation with worse prognosis in eCCA.	[53]
	COX-2 upregulation	Promotion of CCA growth and invasion.	[54,55,56,57,58]
Protein metabolism	Glutamine depletion	Strong depletion: induction a cessation of proliferation or cell death (in vitro addiction to glutamine). Gradual reduction: eCCA cells (EGI-1 and TFK-1) could proliferate under long-term glutamine withdrawal overcoming their addiction to glutamine.	[59]
	ASS deficiency	Reduction of arginine in the surrounding tumor cells could lead to a reduction in CCA cell proliferation.	[60]
	LAT1 overexpression	Activation of mTOR pathway thus affecting cell proliferation and viability.	[61,62,63]
Iron Metabolism	TfR1 high expression	Contribution to CCA progression and poorer clinical outcomes.	[64]
	Ferritin high expression	Negative prognostic index for CCA patients.	[65]
	Fpn reduced mRNA levels	Reduction of iron release, in tumor cells of CCA patients sample compared to matched surrounding liver, suggests that elevated iron content is a negative prognostic factor.	[65]

**Table 2:** Metabolic targeted therapy in CCA.

Target	Glucose Metabolism	Reference
mTOR	Everolimus	[104,105,106,107]
	Sirolimus	[108,109]
	Everolimus + CisGem	[110]
PI3K	LY294002	[111]
	Buparlisib	[112]
	PI-103	[113]
	Buparlisib + mFOLFOX6	[114]
	Copanlisib ± GemorCisGem	[115]
AKT	MK2206	[116]
IDH1	AG-120	[117]
	AG-221	[118]
	AGI-5198	[48]
	**Lipid Metabolism**	
SPHK2	ABC294640	[100,101,102,103]
HMGCR	Simvastatin, Atorvastatin	[119]
	**Protein Metabolism**	
LAT1	2-aminobicyclo-(2,2,1)-heptane-2-carboxylic acid	[62]
	JPH203	[63]
ASS	ADI-PEG 20	[120,121]

## Abbreviations

Cholangiocarcinoma (CCA), cancer stem cells (CSCs), peroxisome proliferator activated receptor gamma coactivator 1-α (PGC-1α), oxidative phosphorylation system (OXPHOS), tricarboxylic acid (TCA), hexosamine biosynthesis pathway (HBP), glucosamine-fructose-6-phosphate amidotransfrase (GFAT), pentose phosphate pathway (PPP), lactate dehydrogenase A (LDH-A, Pyruvate dehydrogenase kinases (PDKs), hypoxia inducible factor α (HIF1α), pyruvate dehydrogenase (PDHA1), phosphatidylinositol 3-kinase (PI3K), signal transducer and activator of transcription 3 (STAT3), nicotinamide adenine dinucleotide (NADH), nicotinamide adenine dinucleotide phosphate (NADPH), peroxisome proliferator-activated receptor (PPAR), free fatty acids (FFAs), fatty acids (FAs), triacylglycerides (TAGs), hydroxy-methylglutaryl (HMG-CoA), reactive oxygen species (ROS), cyclooxygenases (COX), prostaglandins (PG), growth factor receptors (GFRs), epidermal growth factor receptor (EGFR), fatty acid synthase (FASN), cluster of differentiation 36 (CD36), fatty acid transport proteins (FATPs), epithelial to mesenchymal transition (EMT), growth factor-beta (TGFβ), tumor necrosis factor-α (TNF-α), L-type amino acid transporter 1 (LAT1), argininosuccinate synthetase (ASS), hepatocellularcarcinoma (HCC), serine synthesis pathway (SSP), farnesoid X receptor (FXR), transferrin receptor (TfR1), ferroportin (Fpn), insulin growth factor receptor (IGFR1), uncoupling Protein 2 (UCP2), nMYC downstream-regulated gene 2 (NDRG2), obeticholic acid (OCA), isocitrate dehidrogenase (IDH), programmed death-ligand 1 (PD-L1), programmed cell death protein 1 (PD-1), platelet isoform of phosphofructokinse-1 (PFKP), intrahepatic biliary organoids (IBOs), hepatocyte growth factor (HGF), (cancer associated fibroblasts (CAFs), Vascular Endothelial Growth Factor (VEGF), Toll-like receptors (TLRs).
